# Using interactive digital notebooks for bioscience and informatics education

**DOI:** 10.1371/journal.pcbi.1008326

**Published:** 2020-11-05

**Authors:** Alan Davies, Frances Hooley, Peter Causey-Freeman, Iliada Eleftheriou, Georgina Moulton

**Affiliations:** School of Health Sciences, University of Manchester, Manchester, United Kingdom; University of Toronto, CANADA

## Abstract

Interactive digital notebooks provide an opportunity for researchers and educators to carry out data analysis and report the results in a single digital format. Further to just being digital, the format allows for rich content to be created in order to interact with the code and data contained in such a notebook to form an educational narrative. This primer introduces some of the fundamental aspects involved in using Jupyter notebooks in an educational setting for teaching in the bio/health informatics disciplines. We also provide 2 case studies that detail how we used Jupyter notebooks to teach non-coders programming skills on a blended Master’s degree module for a Health Informatics programme and a fully online distance learning unit on Programming for a postgraduate certificate (PG Cert) in Clinical Bioinformatics with a more technical audience.

## Introduction

Universities and other higher education (HE) institutions are now under increasing pressure to provide more online and distance learning courses and to deliver them cost effectively and rapidly [1]. This increase in demand is partly based on students wanting more flexible study options in comparison to traditional HE course delivery to aid in study around employment and family commitments. This is also driven by financial considerations that allow HE institutions to scale course delivery while managing infrastructural provision (e.g., access to rooms for teaching and limited capacity for face-to-face delivery) [2]. To meet this challenge, we require tools that cater for students with varying levels of digital literacy and reduce the burden of them having to download and install software, all of which requires support, which is more difficult to provide at a distance. This can be further complicated when students use managed equipment (e.g., National Health Service (NHS) employees) and may not have administrator rights to install software.

Digital notebooks provided us with a way of meeting these needs as they are easy to set up, straightforward to use, and can support rich and interactive content. Here, we present a primer on how to use digital notebooks (specifically Jupyter notebooks) for teaching and assessment along with details of 2 case studies where we used notebooks to teach Python programming and database skills for Clinical Bioinformatics and Health Informatics students of varying levels of technical experience. The case studies and methods presented can be applied to both distance learning and face-to-face teaching scenarios.

We will start by covering what a Jupyter notebook is along with the different “cell” types available. We then look at how they can be run and enhanced with extensions to add items like exercise tasks and other interactivity before looking at how they can be used in assessment. Next, we present 2 case studies where we have applied notebooks to teach different groups of students to give some examples of the different contexts they can be used in. Finally, we end with a discussion to synthesise our experiences of using notebooks to educate students and their further potential with considerations for education.

### What is a Jupyter notebook?

**Fig 1:**
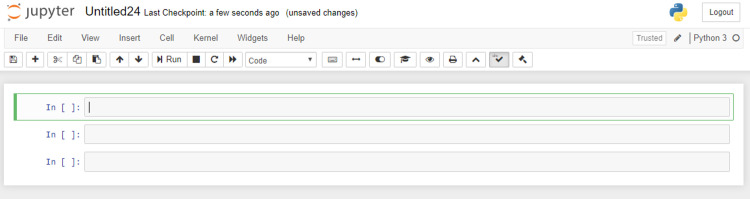
A new Python 3 notebook with 3 empty cells denoted by the grey rectangles. The currently selected cell is highlighted in green.

Jupyter notebooks are an open-source web application that run in an internet browser. They allow the sharing of code, data analysis, visualisations (which can be interactive), math formulas, and other embedded media (e.g., YouTube videos, images, and web links), all in a single document combining interactive and narrative components. This takes the form of a document that is composed of multiple cells that encapsulate the content of the notebook (Fig 1).

Jupyter notebooks were created by Project Jupyter, which, according to their website, states that “Project Jupyter exists to develop open-source software, open-standards, and services for interactive computing across dozens of programming languages.” [3] This includes various standards for interactive computing, including the notebook document format that is based on JavaScript Object Notation (JSON). The name Jupyter is composed of the initial 3 languages supported: Python, Julia, and R [4].

### Anatomy of a notebook

**Fig 2:**
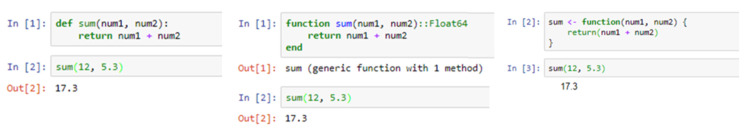
A simple function that returns the value of the sum of 2 numbers showing different kernels (programming languages) in the notebooks. (Left) Python, (middle) Julia, and (right) R.

Jupyter notebooks are available in various programming languages with current support for over 40 different programming languages [3]. These include the popular languages used for data science, such as Python, R, and Julia (Fig 2).

The notebooks are made up of units called “cells” that can be executed (run) in order to render their contents in different ways.

### Cell types

There are 2 principle cell types. The first cell type is the “**Markdown”** cell, which is used to present text, images, equations, and other resources. The second cell type is the “**code”** cell that allows the user to enter code written in a chosen programming language that will execute in the notebook. To execute the contents of any cell, the user can press the SHIFT and ENTER keys together or alternatively click on the “**Run”** button in the main menu bar across the top of the screen. If the cell being run is a code cell, it will cause the code in the cell to be executed and any output displayed immediately below it. This is indicated by the “**In”** and “**Out”** words located to the left of the cells as seen in Fig 2.

### Styling cells

**Fig 3:**
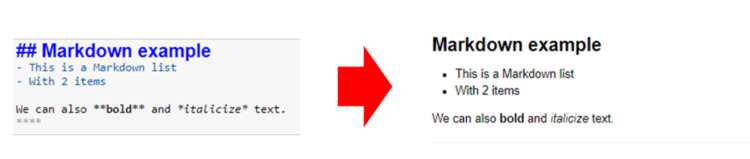
Example of a markdown cell (left) and the output of the styled cell when the cell is run (right).

Markdown cells can be styled with Markdown, which is a lightweight mark-up language for styling text [5]. This works by turning Markdown text into HTML (Fig 3).

**Fig 4:**
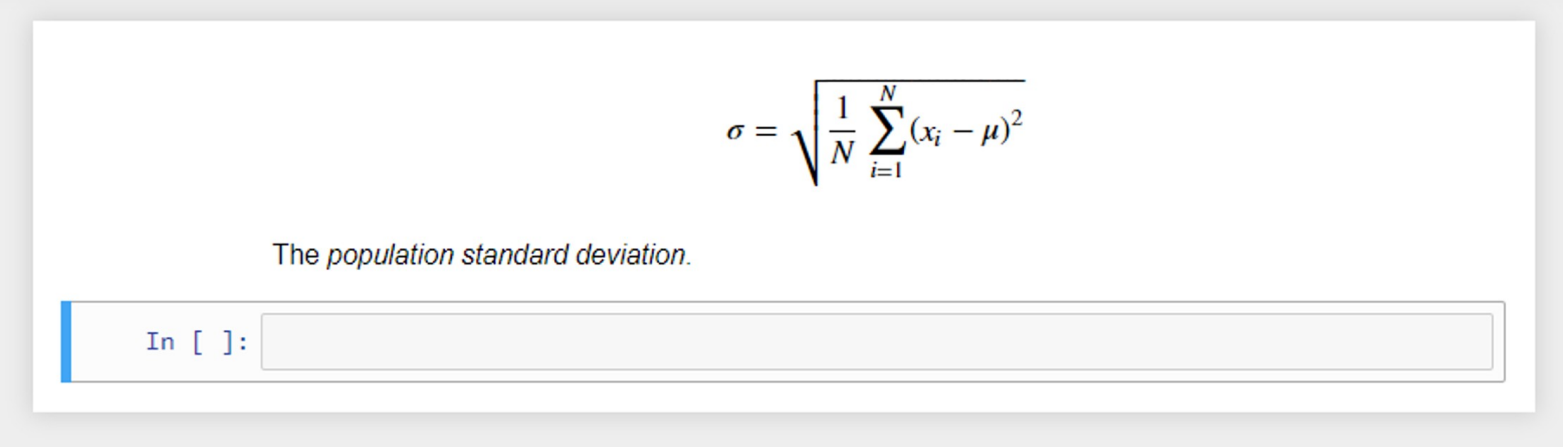
Output of LaTeX math notation producing the formula for the population standard deviation.

These cells can also display plain text as output with no styling. Another useful feature for teaching math-based courses or sharing formulas, etc. is the integration of LaTeX support. LaTeX is a popular typesetting document preparation system [6] that was built on the Tex typesetting language originally developed by the American computer scientist Donald Knuth [6]. LaTeX is widely used by the scientific community (e.g., computer scientists) to write academic publications (journal and conference papers). LaTeX math notation can be added to markdown cells to display formulas using common math notation. For example, the code here produces the output seen in Fig 4.

$ $

\sigma = \sqrt{\frac{1}{N}\sum_{i = 1}^{N} (x_i-\mu)^2}

$ $

The LaTeX wikibook math section [7] is a useful resource for learning about the math notation options available in LaTeX. Table 1 provides an overview of some of useful Python libraries for numerical and scientific computing that can be incorporated into the notebook environment.

**Table 1:** Some useful Python libraries for numerical and scientific computing.

Module name	Description	Link
Pandas	For data analysis; supports objects like dataframes	https://pandas.pydata.org/
NumPy	For scientific computing; supports matrices and arrays	https://numpy.org/
SymPy	For symbolic maths; can also convert Python code into math notation	https://www.sympy.org/en/index.html
matplotlib	Produces publication-quality plots/graphs	https://matplotlib.org/
scikit-learn	A machine learning algorithm library	https://scikit-learn.org/stable/

### Running Jupyter notebooks

**Fig 5:**
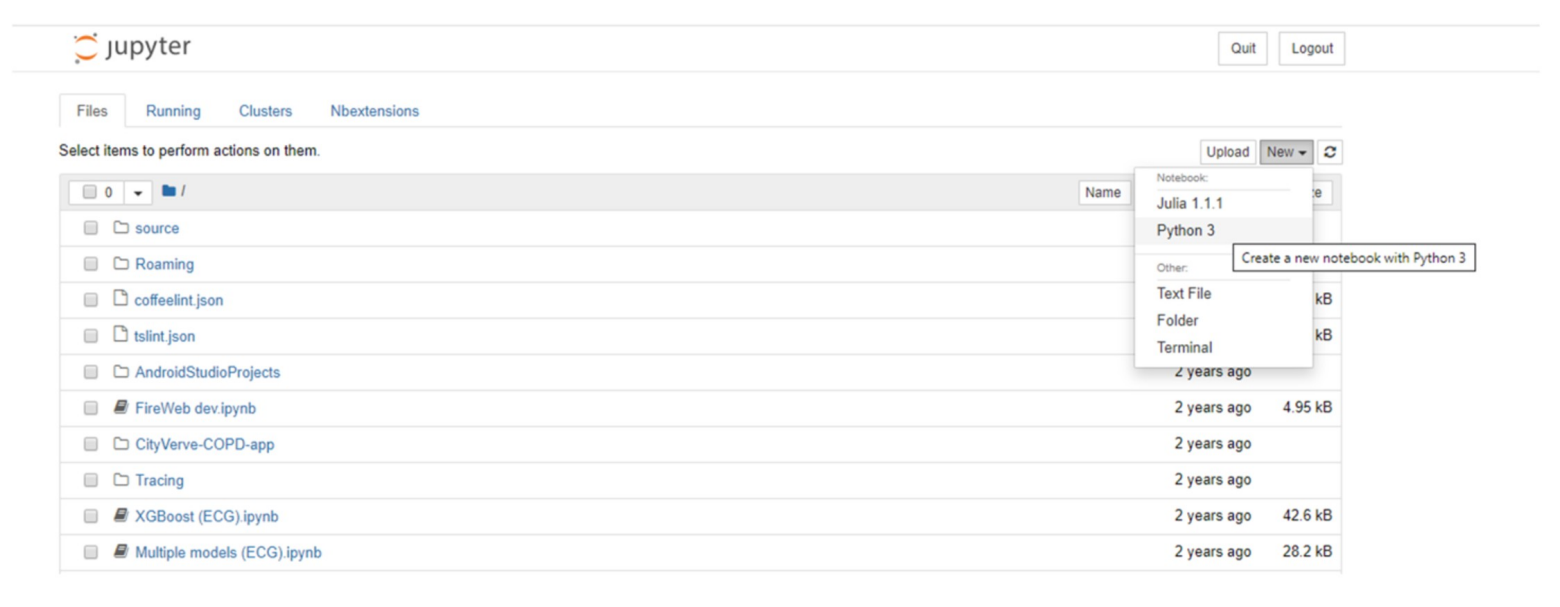
The files and folders tab seen when launching Jupyter notebooks locally. A new notebook is created by selecting the ***new*** dropdown option and choosing the required language.

There are different ways of accessing Jupyter notebooks. The Anaconda distribution [8], a Data Science platform for Python and R, provides a free Python distribution, which includes Jupyter notebooks. Other options include JupyterHub [9], which is designed for groups of users to access notebooks on the cloud or locally hosted and maintained on their own devices. Once run, the user is greeted with a page showing the various files and folders available (Fig 5). Selecting the “**new”** option from the menu allows the user to create a new notebook in the selected language; alternatively, an existing notebook (ipynb) file can be loaded by selecting the required file from the list of files in the main list to the left of the screen.

### Jupyter Notebooks, JupyterLab, and JupyterHub?

Project Jupyter has created several resources and services surrounding the initial notebooks. This can sometimes cause some confusion among beginners. A brief description includes the following:

- Jupyter Notebooks: an interactive computational web application that combines code, text, data analysis, and other media in a single document;
- JupyterLab: builds on the original notebooks to provide an online interactive development environment that allows users to access notebooks with data and file viewers, text editors, and terminals all in the same environment. This helps to better integrate notebooks with other documents and resources in a single environment; and
- JupyterHub: let multiple users (groups) access notebooks and other resources. This can be useful for students and companies that want a group(s) to access and use a computational environment and resources without having to install and set things up. The management of which can be carried out by system administrators. Individual notebooks and the JupyterLab can be accessed via the Hub. The Hub can be run in the cloud or on a groups own hardware.

As these offerings build on the initial notebook and have notebooks at their core, this article describes the notebooks for beginners, rather than the additional platforms and services that incorporate them. Notebooks themselves work in a similar way regardless of being accessed alone or via JupyterLab or JupyterHub. It is worth being aware of these options, however, for building and sharing resources around the notebooks that you may develop.

### Notebook extensions

A number of different “bolt on” extensions exist for the notebooks. These can be extremely useful for including additional features into a notebook. Some examples include the ability to split a cell into 2 different cells horizontally, a spellchecker, auto numbering of equations, and an extension for making exercise tasks (discussed later). To utilise the additional features that are available with the notebooks, the following commands (Box 1) should be entered into the command prompt (e.g., the Anaconda prompt or Powershell):

**Box 1. Commands to enable notebook extensions**

pip install jupyter_contrib_nbextensions

jupyter contrib nbextension install—user

pip install jupyter_nbextensions_configurator

jupyter nbextensions_configurator enable–user

**Fig 6:**
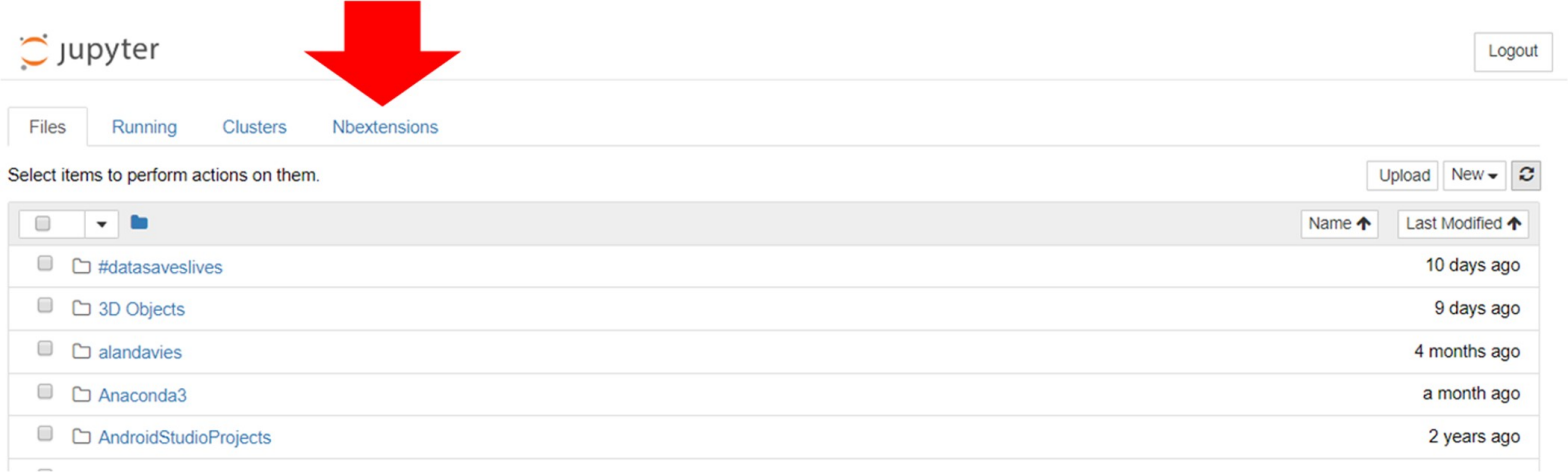
The NBextensions tab for selecting the various notebook extensions.

This enables the “NBextensions” tab (Fig 6). When clicked on, the user is presented with a series of checkboxes for the various extensions. There is also a description, often with associated screenshots and/or animations previewing what the extension does.

**Fig 7:**
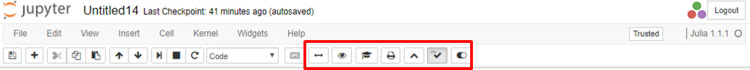
Enabled notebook extension icons shown in red box.

When a new notebook is opened, the selected extensions appear as small icon buttons under the main menu (Fig 7).

### Magic commands

IPython (the “Interactive Python” kernel used in Jupyter notebooks) also supports what are known as magic commands or functions, which are used to change the standard behaviour of IPython. Magic commands come in 2 different types: “**line**” and “**cell”** magic’s (Box 2).

**Box 2. The first line is magic to list all the available line magic’s. The second line displays a help window with information about magic functions**

%lsmagic

%magic

**Fig 8:**
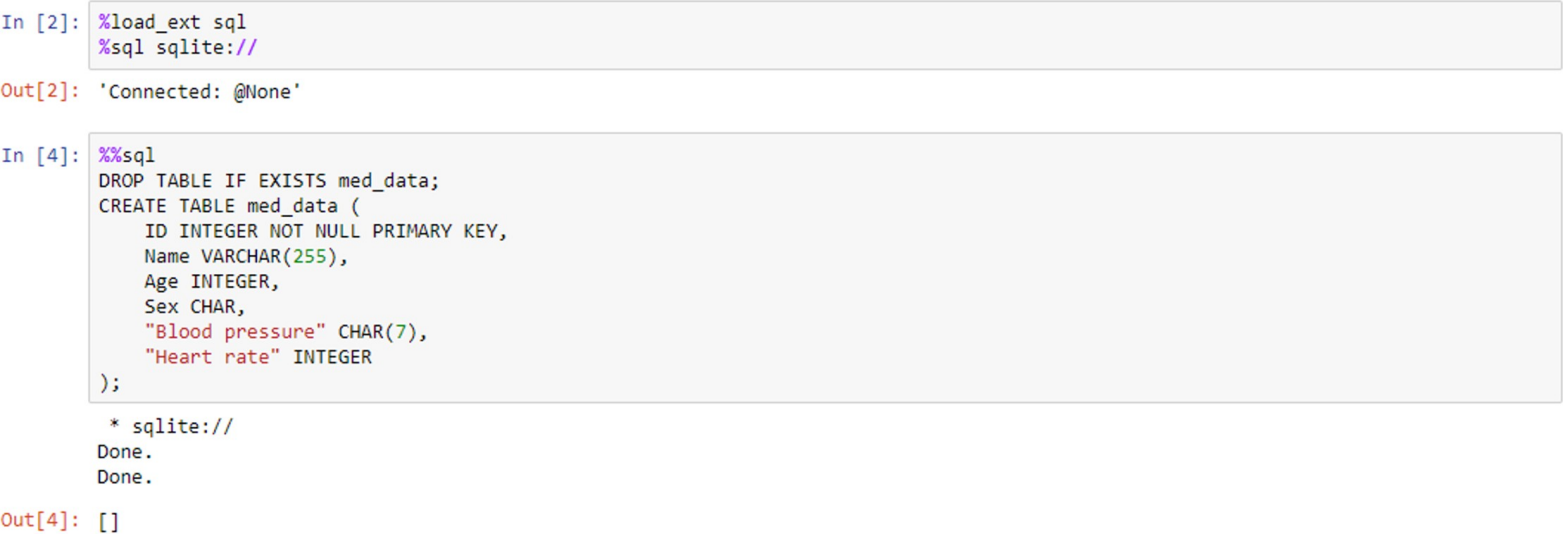
Line and cell magic’s used to add SQL functionality to a Python notebook. SQL, Structured Query Language.

Line magic works on the line of code that it precedes only, whereas cell magic applies the function to the entire cell. Line magic is prefixed with a single percentage character (%), cell magic with 2 percent characters (%%). Fig 8 shows an example of this, where we use the magic functions to load a Structured Query Language (SQL) extension and specify the database engine such as SQLite. The second code cell employs cell magic to allow us to write and execute SQL commands in the notebook environment to create a database table.

### Widgets

**Fig 9:**
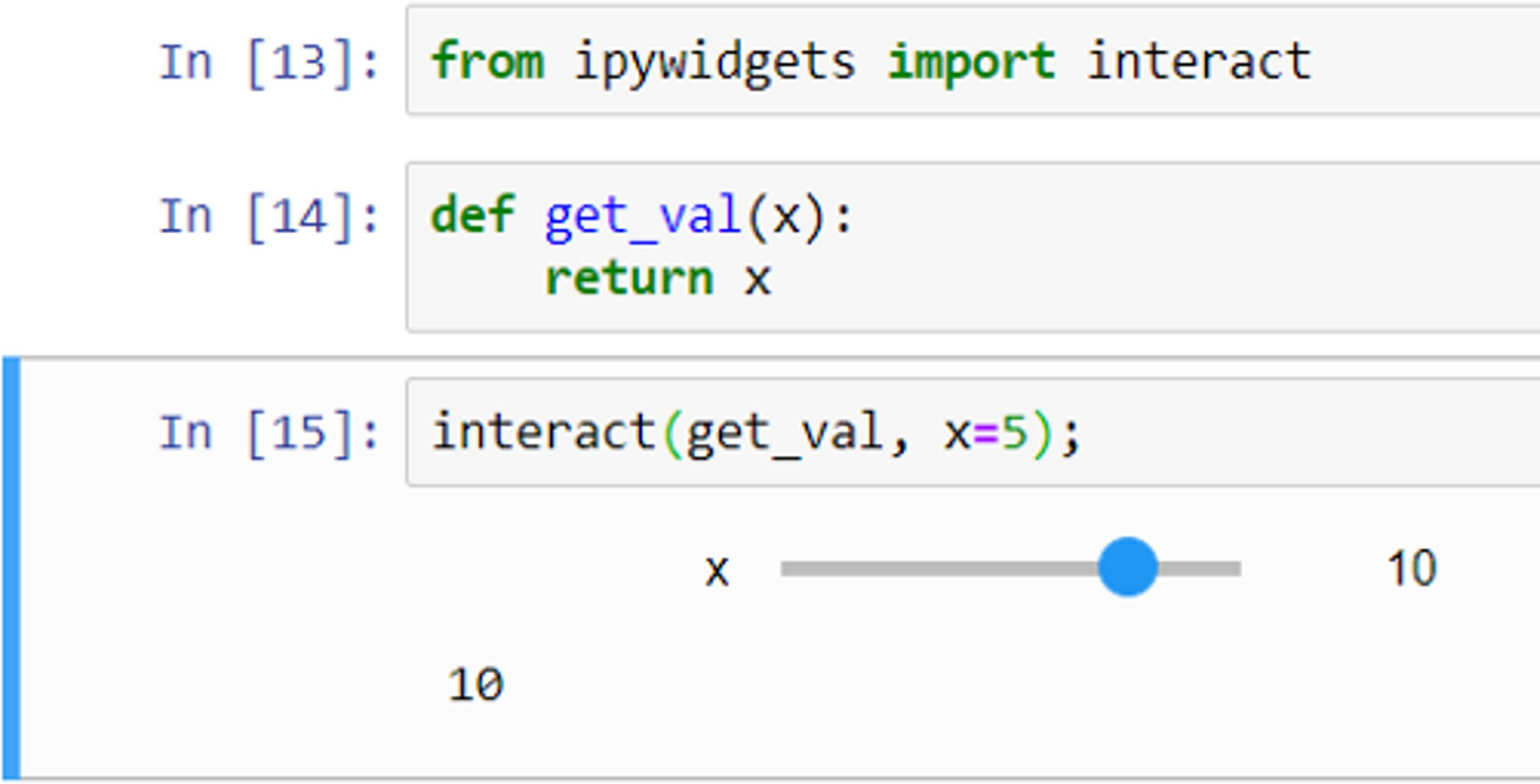
Example of notebook interaction.

**Fig 10:**
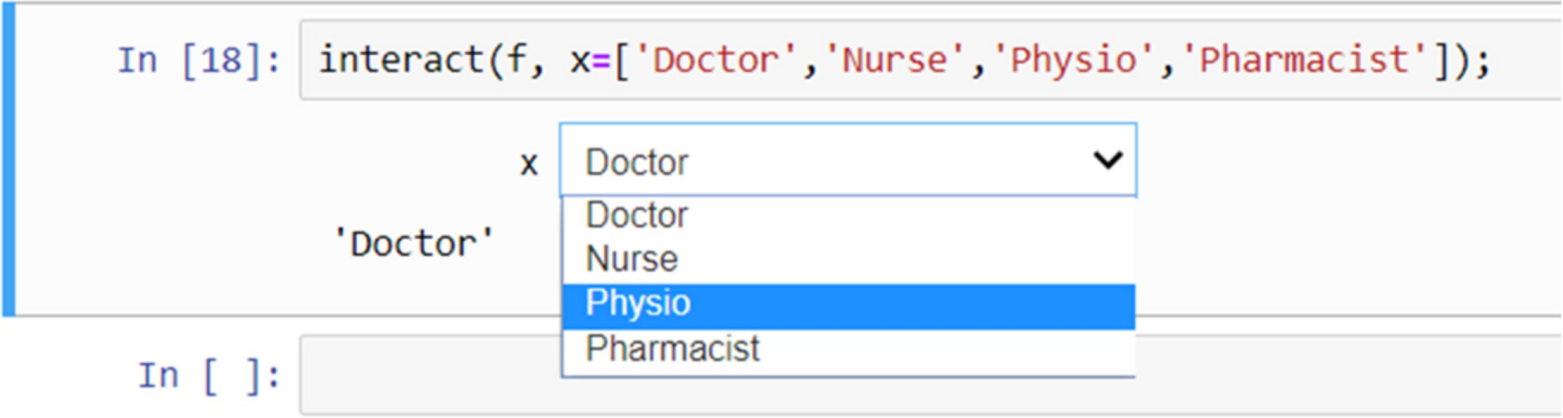
An interactive drop-down list created using a Python list.

Widgets can be used for interactive elements in notebooks [10]. Fig 9 shows an example of this where the “**interact**” function runs the “**get_val**” function displaying a slider with the default value (5 in this case) selected. The user can then change the value by moving the slider to the left or right. Fig 10 shows another example, this time using a drop-down list of options created from a Python list.

A more substantive example of using interactive widgets is highlighted by Richardson and Behrang who use Python notebooks to view Digital Imaging and Communications in Medicine (DICOM) images [4].

### How Jupyter enhances collaboration and reproducibility

Reproducibility in science is an important concept. Without which, there is a lack of transparency about what was done. One would expect that if scientists follow the same method, the results will be the same. This is sometimes difficult to achieve with complex data and analysis methods. The quality of research in relation to collaboration was brought into question in a recent Wellcome Trust report on research culture that stated there was some concern over the impact of lack of research collaboration on research quality, and in some cases, unhealthy competition between researchers [11]. Hardwicke and colleagues highlights that the availability of data is essential for a self-correcting ecosystem in science and that this can be undermined by unclear analysis and poorly curated data, which, in turn, impedes analytic reproducibility [12].

There has been a counter movement to improve these issues with organisations such as the UK Reproducibility Network (UKRN) [13], which is a network of 10 universities in the UK that are concerned with reproducibility in research. The founder of UKRN calls for institutional changes to promote open-research practices [14]. Although various research studies do share their data, other researchers’ understanding of the shared dataset and their ability to repeat the previous analysis hinges on the documentation of both the dataset and analysis steps followed, as well as being able to replicate the software environment in order to run the code in the first instance. Because of these requirements, notebooks are being used increasingly by researchers to share analysis code along with an explanation and steps involved in processing the data for reproducible research purposes. This has led to wide-scale use in the research community [15]. By using interactive notebooks, the data analysis code and steps taken can be shared together with any additional documentation, formulas, etc., that are required to understand the applied method. Sharing data and analysis code in such a way dramatically improves the speed in which the analysis can be rerun by other researchers. Researches are also building on notebook technology for novel purposes, for example, Tellurium notebooks that were developed to support the creation of reproducible models for systems and synthetic biology [16].

**Fig 11:**
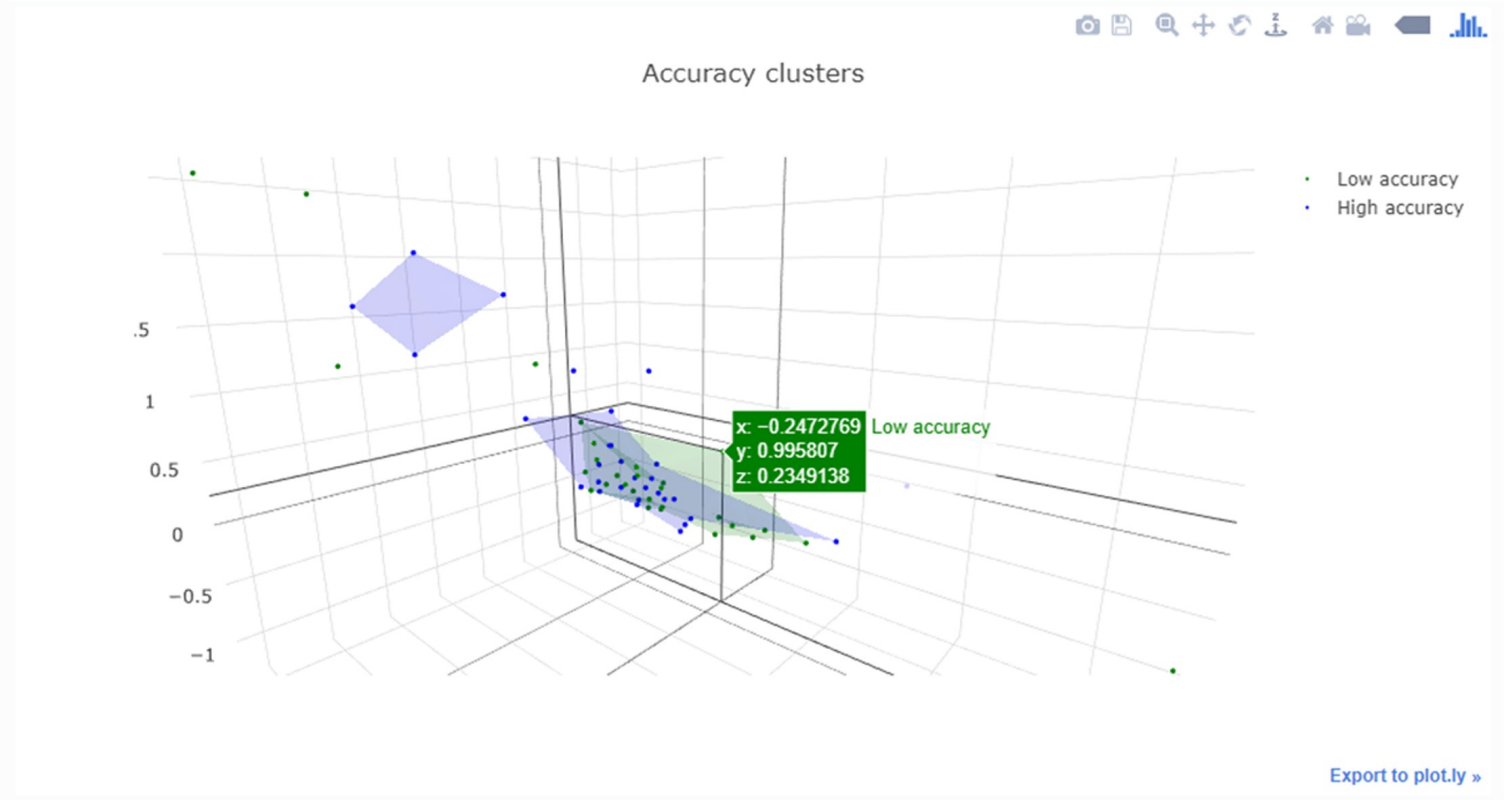
Interactive plot generated with the “plotly” module that can be rotated and zoomed with individual data points selected.

Aside from the research applications, Jupyter notebooks are also being increasingly used to teach subjects like data science and programming [17] as they feature dynamic responses such as interactive visualisations and rapid updating of results based on the filtering of data (e.g., Fig 11).

### Notebooks and assessment

Notebooks can also be set up to carry out formative or summative assessment. The “**nbgrader”** tool [18] allows for the creation and grading of assignments in the notebook environment. The tool allows a user to generate an instructor version of a notebook that has predefined solutions. This, in turn, is used to generate the student version of the notebooks without the solutions. These student versions of the notebook(s) can then be distributed to the students by email or via a virtual learning environment (VLE). The principal aims of the tool were to address issues surrounding the maintenance of separate student/instructor notebook versions, automatic grading of exercises, the manual grading of “free response” questions, and the ability to provide feedback to students. There are 2 ways of using the nbgrader. The first is a standalone version; the second is designed to work with JupyterHub, which can manage the release and collection of submitted assessments. The nbgrader adds a tool bar to each cell to make the cell either an “**answer”** or a “**test**” cell. The answer cells allow students to add code placed between a placeholder. Unit tests are written by the instructor to evaluate the correctness of the student’s solution. Tests can also be hidden from the students. Points can be assigned to each cell to assign specified marks if the unit tests pass. Cells can also be set to “**manually graded**” answers so students can write free text, code formulas, etc. Student feedback can be provided when grading by adding text to any required cell and then converting the notebooks into HTML format so they can be emailed/added to VLE for the students to view.

**Fig 12:**
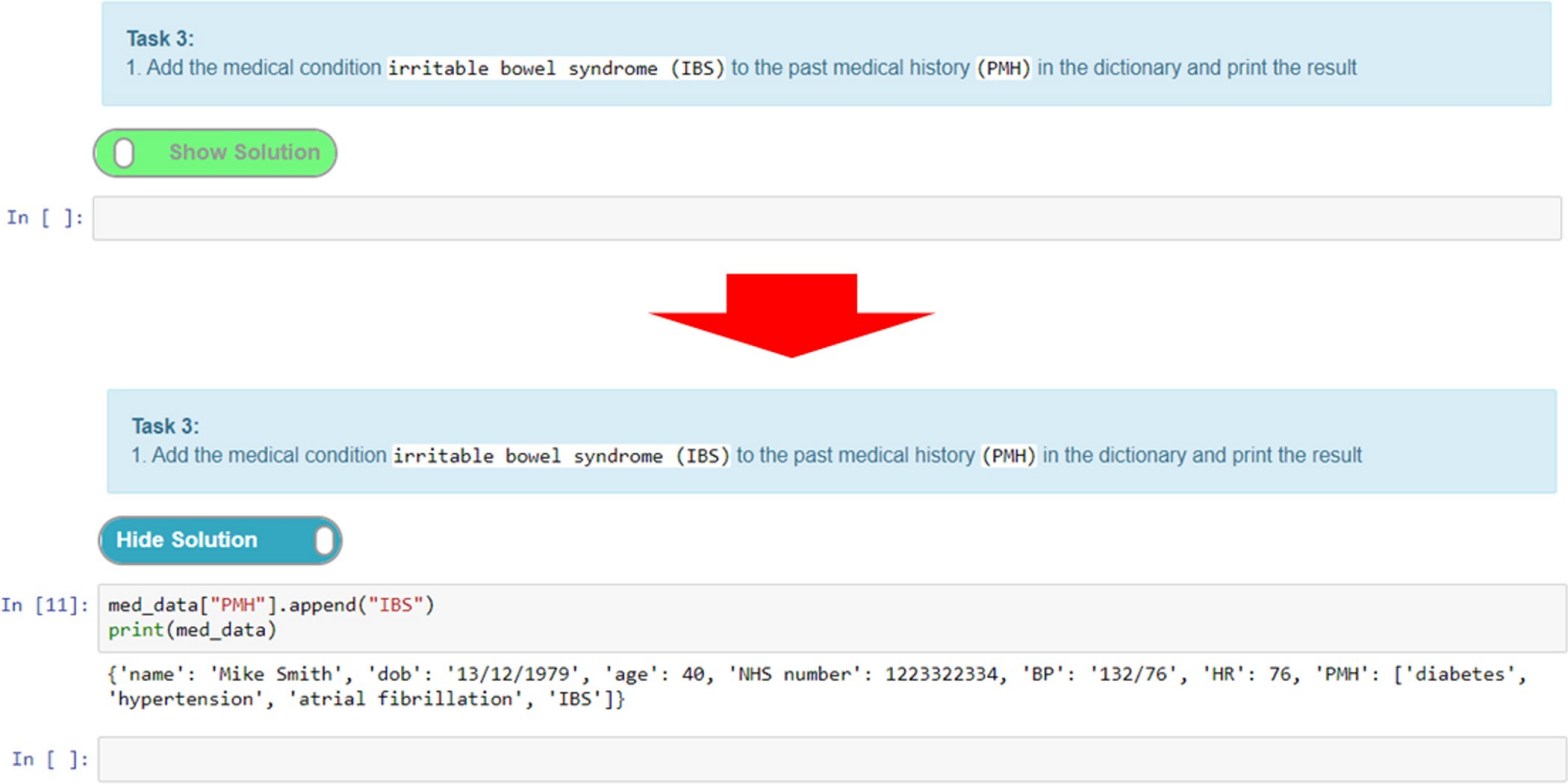
Example using the “exercise2” extension to create a task. When the “show solution” button is pressed, the answer is displayed below.

A simpler method of providing interactive tasks for formative assessment that does not require the knowledge of writing unit tests is to use the exercise extension. This extension can be used to add exercises (Fig 12). Adding feedback in the form of exercises is a unique feature of notebooks that elevates them from simply being an online textbook. The ability to provide interactive tasks that let students engage directly with the notebook without the need to use additional software is a powerful feature. Moreover, this helps maintain the narrative flow, as the exercises can be woven into the content in appropriate places without diverting the user to other tools or resources, all of which helps with the overall user experience.

Here, we give an example of creating an exercise where we create a task cell and put the solution in the preceding cell. The solution cell can be hidden until the “show solution” button (Fig 12) is activated, which reveals the hidden cell. This is a good way of adding coding tasks for students and then presenting them with a model answer/solution for comparison and/or further explanation. Fig 12 shows a task where the student has to add a textual value to a Python dictionary data structure and output the result. Students can attempt to write the code for this and then toggle the solution to check their answer with the one provided.

### Sharing your notebooks

Notebooks can be shared in the same way as any other file. In order to run a notebook, however, users will need to install and set up software (i.e., Anaconda). This may not be the ideal solution given that novices may have difficulties installing and setting up the environment required to view and use notebooks. This is further compounded if the user needs to install extra libraries and extensions that may be required to run a notebook as intended. One way around this that is helpful when sharing notebooks with novices is the Binder Project [19].

Binder is a web service (currently open source) that allows users to create interactive sharable and reproducible computational environments in the cloud [20]. Binder uses several different technologies (i.e., repo2docker, JupyterHub, and BinderHub) that allow a user to place their notebooks in a repository (e.g., GitHub). Once done, a form can be filled in on the Binder website (mybinder.org). This includes a repository Uniform Resource Locator (URL), Git tag, and optional path of notebook file. Following this, a user will receive a URL that they can send to others to share their notebooks.

For a more technical explanation of how Binder works, please see the Binder paper presented at the SCIPY conference in 2018 and its associated YouTube video [20]. For more information on how to implement sharing Notebooks with Binder, see the Data Carpentry tutorial that guides users through sharing their notebooks with GitHub and using Binder [21].

## Case studies

We present 2 short case studies detailing how we have used Jupyter notebooks to teach programming skills to different audiences on 2 of our courses, an MSc module on a Health Informatics programme and an introduction to programming module on postgraduate certificate (PG Cert) in Clinical Bioinformatics. This is followed by a brief initial evaluation of the use of notebooks in our teaching.

### Case study 1: Modern Information Engineering (MIE)

**Fig 13:**
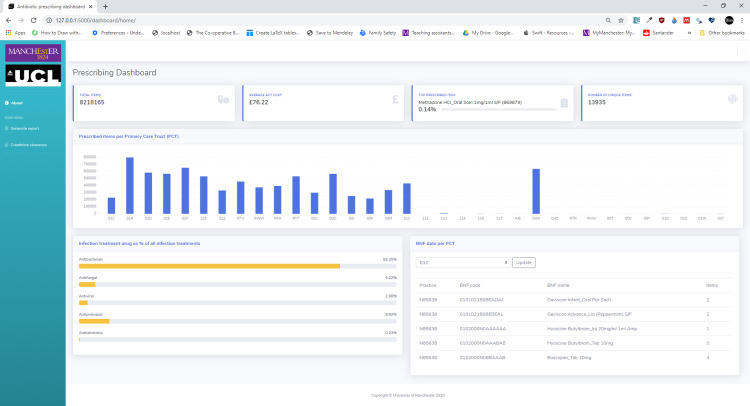
Example of the prescribing dashboard the teams would add functionally to following the Scrum framework.

The Modern Information Engineering module is a new 15-credit master’s level optional course unit that was proposed to model the process of modern software development using the Scrum framework [22] from the Agile software development methodology [23]. The unit was delivered in a blended format with both distance learning and a 3-day block of face-to-face teaching sessions. Students (*n* = 21) were from a variety of backgrounds. Nine (43%) were NHS Graduate Management trainees. A further 4 (19%) had clinical backgrounds. The rest (38%) had a variety of backgrounds. The course runs over a 9-week period with students working in Agile teams to add functionality to a medication prescribing dashboard (Fig 13) written in Python using the Flask web framework [24]. Students work in Sprints (2-week cycles) to add features to the dashboard, of which the skeleton code was provided to the groups.

**Fig 14:**
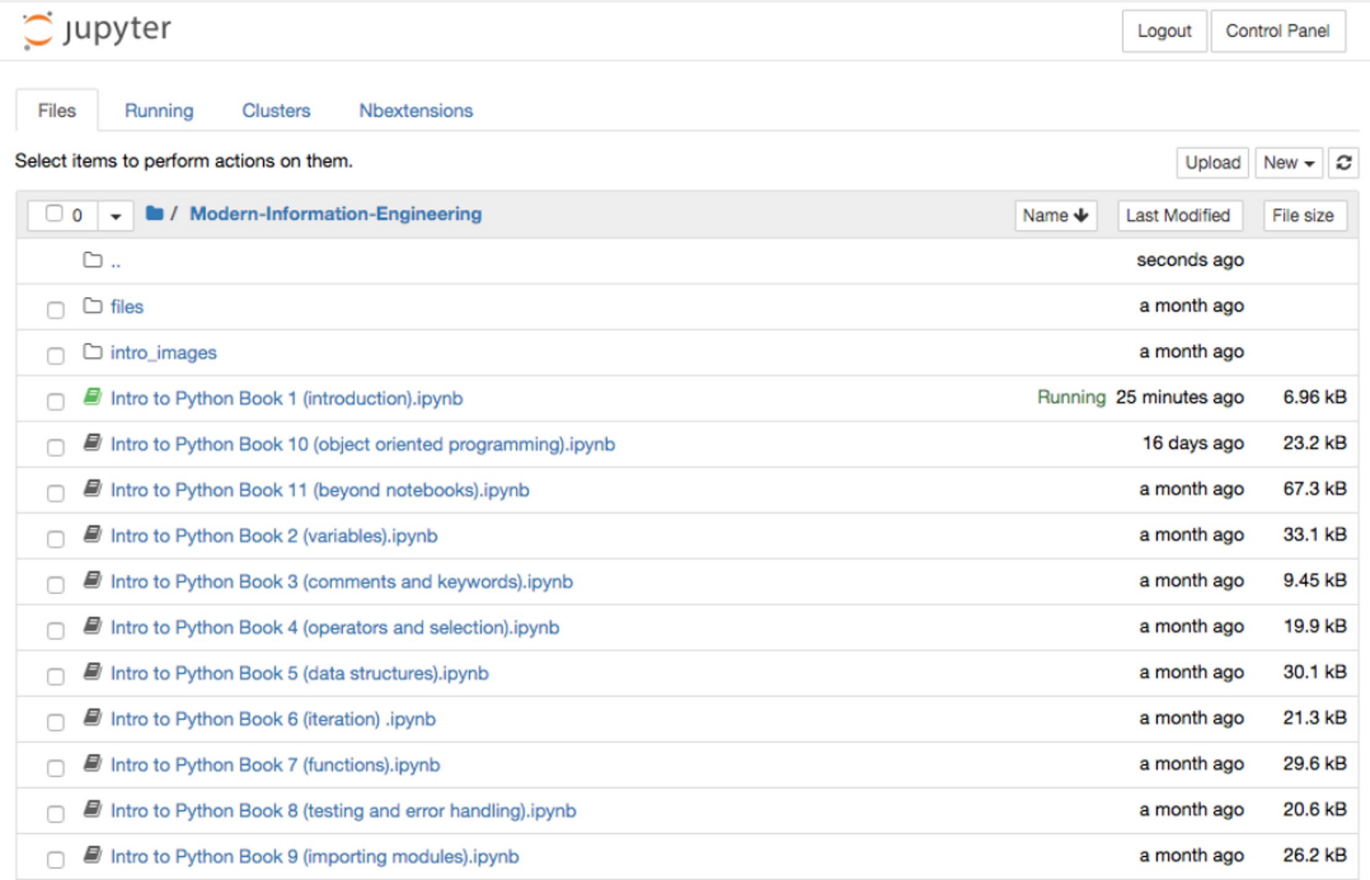
List of notebooks covering the various topics of programming with Python.

The first part of this unit involved teaching fundamental Python and databases skills using SQL to students, many of whom have had no or limited exposure to computer programming (coding). We implemented the teaching of Python and SQL in the Jupyter notebook environment. The unit was a module available as part of the MSc Health Informatics programme that is a joint award between the Universities of Manchester and University College London (UCL) [25]. The principal challenge faced was delivering teaching of coding skills to those who have little or no coding experience via distance learning in a way that allowed them to focus on obtaining these fundamental skills in the chosen language (Python) without introducing any additional complexity to the process. A further challenge was that unlike undergraduate courses where we may teach in person using a PC cluster (computer room/lab) with preloaded software managed by IT services, many master’s level students will be required (and usually prefer) to use their own computing devices (laptops/tablets/desktops). Supporting the use of software on these different operating systems and platforms adds an additional challenge. In order to remove or reduce these barriers to learning, we decided to make use of the interactive Jupyter notebooks that support among others the Python programming language. We were then able to host a set of notebooks taking students through the various coding topics in order (Fig 14). A link to the notebooks was provided on the VLE for the module (i.e., Moodle) and the universities central username and password system added to prevent non-university affiliated personnel accessing the notebooks.

**Fig 15:**
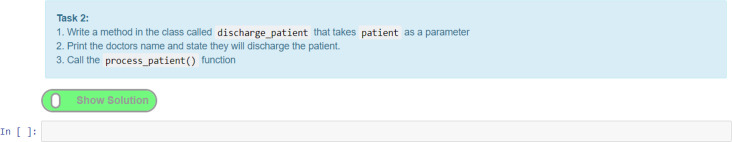
Example of task from notebook. Clicking the “Show Solution” button reveals the model answer.

The main reason we built our own notebooks rather than link to other existing resources (e.g., Software Carpentry, https://software-carpentry.org/) was to provide specific health-related examples for the students so that the domain would be familiar to them. Many of the computer science examples can be abstract in nature. By providing concrete health examples, it was hoped that this would help the students to see the relevance of potential applications of programming in health settings. We were also able to add tasks throughout the notebooks that allowed students to code in the notebook and then view a model answer (e.g., Fig 15) using the exercise extension discussed previously.

**Fig 16:**
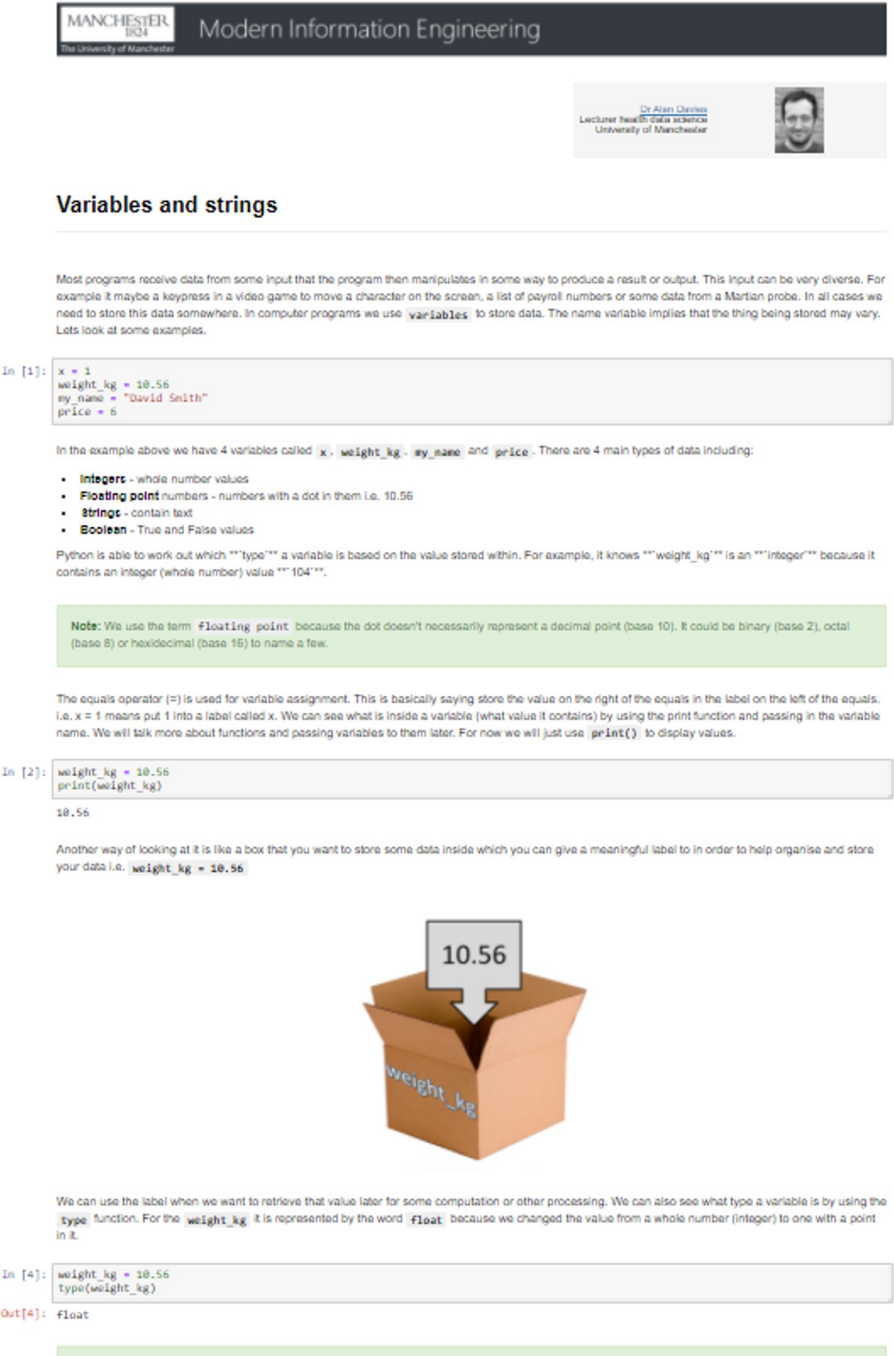
Example of notebook on variables and strings programming topics.

Fig 16 shows an example of a notebook from the set about the topic of variables and strings.

Using the notebooks in this way allowed us to side step the issues of asking the students to download and set up Python on their machines with the associated complexities of supporting this. We do this later on in the unit where we move to group work and using an integrated development environment (IDE). At the beginning of the unit, we remove this barrier and allow the students to focus on learning the Python language and programming fundamentals. Initial feedback suggests that this improved their confidence with coding prior to the summative portion of the module. We provided support for students using the notebooks through the VLE and also by Slack (a cloud-based instant messaging service). Teaching assistants (TAs) would monitor the Slack channels and respond to issues the students faced with running the notebooks and Python in general.

### Case study 2: Introduction to programming

A second case study involving the teaching of basic coding in Python was a 15-credit module in a new distance learning PG Cert in Clinical Bioinformatics. It was designed to teach the fundamentals of genomics medicine to a diverse cohort of students. Clinical Bioinformatics is a relatively new profession and represents the marriage of computer science with clinical practice. The computational and data skills needed to become a clinical bioinformatician are in short supply in the NHS with training and education trying to fill the skills gap [26]. Those new to the field could come from many backgrounds, for example, those from the health sector with little or no programming experience to those with IT knowledge but with limited clinical experience.

Similarly, to case study 1, the module also adhered to agile principles [23] but was delivered entirely online. The first part of the unit involved teaching basic GitHub and Python skills to students with differing levels of programming experience. It needed to support these varied learning requirements but also support students remotely, without face-to-face contact while emulating clinical bioinformatics in practice. We therefore created an immersive and realistic software development environment with real-world practice-based problems in the form of sprints. To ensure an authentic learning experience, the students were taught to use Anaconda to install Python 3 onto their own machines. They also installed Git, and Windows users also installed and initialised Git Bash so that all students could be taught in a LINUX environment. The course content was delivered outside of Blackboard (learning management system) to the students using GitHub (https://github.com/i3hsInnovation).

Other than initial introductory materials, the course material was taught using Jupyter Notebooks. The notebooks allowed us to provide interactive teaching on the basic principles of Python programming including exercises that the students could complete within the notebook to hone their skills. Once the basic principles of Python programming were covered, we introduced the students to representational state transfer (REST) APIs commonly used to collate genomics data. The immersive nature of the notebooks allowed us to build authentic tutorials to help students understand how data are retrieved from REST APIs and how they could build their own REST APIs. The notebooks gave the students the space to practice and develop these new skills comfortably in a fail-safe environment while using real-world examples. The flexibility of the notebooks also meant we could reuse them easily and incorporate slightly different examples to support the diverse student cohort.

The notebooks introduced the team-based sprint scenarios requiring the students to prototype code that will meet real-world needs of NHS scientists and an in-production genomics software application (VariantValidator, https://onlinelibrary.wiley.com/doi/full/10.1002/humu.23348). The interactive and engaging teaching provided by the notebooks helped scaffold the learning with short snippets of interactive code. These blocks of learning eventually culminated in a final SPRINT project where the learners built resources based on needs from their own practice (or became additional prototypes to support the VariantValidator project).

Other tools such as Slack helped with the group work and educational support, such as solving initial configuration issues, pastoral support, and providing personal feedback on SPRINT activities. This peer-supported learning approach helped hone another essential skill in clinical bioinformatics—being an active member of a community of practice [27]. It was the dual approach of active learning materials providing a fail-safe environment in the notebooks coupled with the peer-supported learning via Slack that meant we were able to deliver effective training into multiple countries including a student working in frontline healthcare in China during the peak of the Coronavirus Disease 2019 (COVID-19) epidemic. At the end of the course, because the notebooks were downloaded to the students’ machines, they had the tools, tutorials, and examples at their fingertips to learn back in practice. The aim of this “sandbox” of editable and authentic learning materials was to help students to strengthen their programming skills in the long term and progress as members of the wider Clinical Bioinformatics community.

### Evaluation

A detailed evaluation is beyond the scope of this paper as we are yet to run the various modules for significant time to collect sufficient data. We do, however, present some initial findings from a survey carried out on units using Jupyter notebooks for teaching as well as some statements from students about their experiences. Twelve students completed the survey and were asked 6 questions concerning the use of Jupyter notebooks. These consisted of the following:

1. How useful did you find this course unit? (1 = not at all, 10 = very useful)
2. How easy was it to use the Jupyter notebooks in your learning? (1 = very difficult, 10 = very easy)
3. Did the notebooks structure and combination of activities help you build understanding? (no/yes)
4. Did the pace of activities feel right to you? (no/yes)
5. How likely would you be to recommend Jupyter Notebooks and the learning approach we have followed? (1 = not at all likely, 10 = extremely likely)
6. Overall how satisfied were you with the course? (1 = least happy, 10 = happiest)

**Fig 17:**
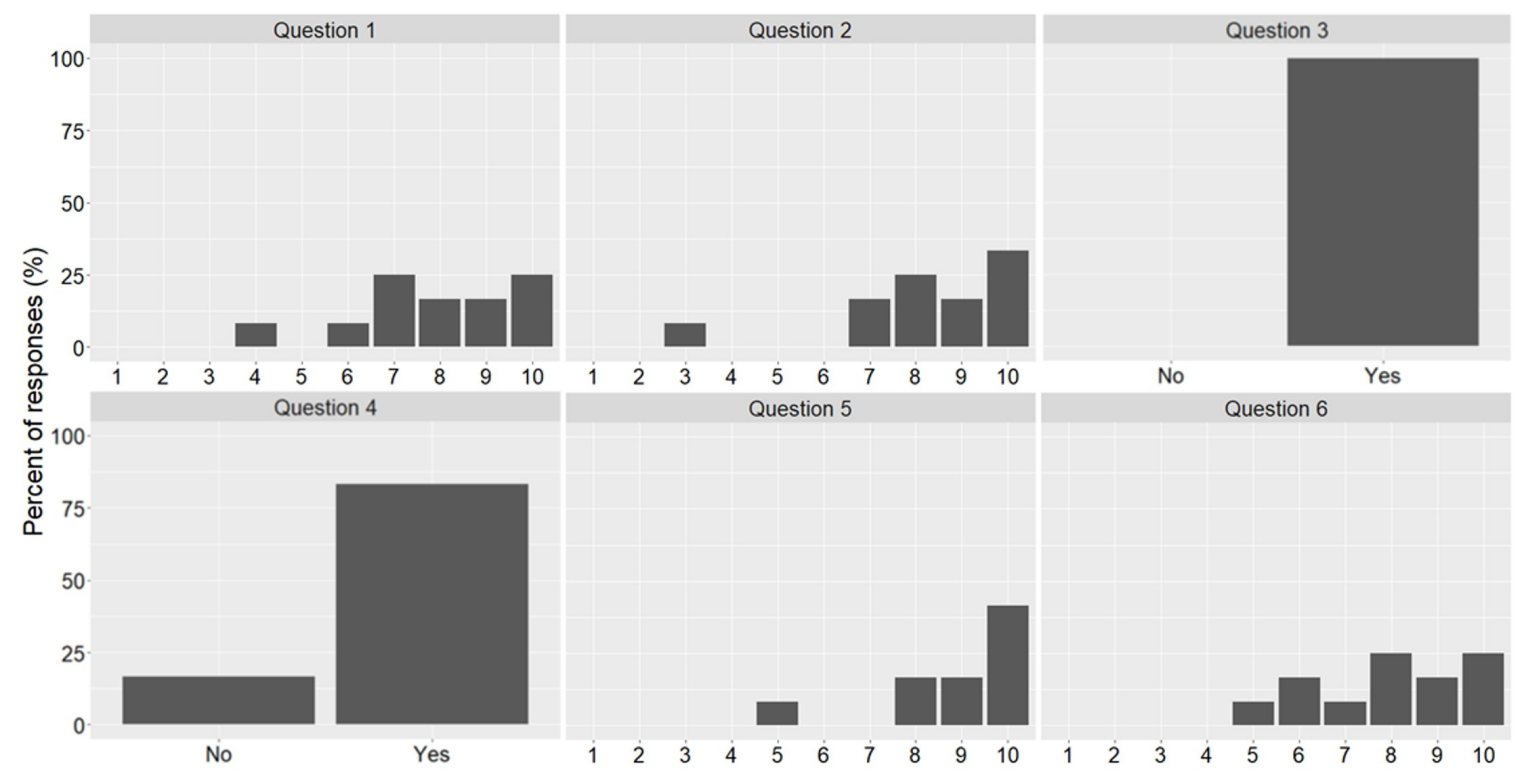
Results of notebook student survey (*n* = 12).

The results of which can be seen in Fig 17. We see that students provided predominantly positive responses to the questions asked. Results show that the students indicated that they would recommend notebooks for learning, found the course unit useful, and were satisfied with the course. For case study 2, students also provided reflective videos and feedback. This included the following comments on the practice-focused Jupyter Notebooks:

“[…] the programming module starts with the basics for students where it is new to them. It gives an excellent overview of the different methodologies and languages and resources that are key to bioinformatics and **what’s also really helpful, or I found helpful, is that the code is taught in snippets in Juptyer Notebooks so you are able to try out small parts of the code for yourself … before you even need to get to grips with the development environment. So that was really useful**”—student on PG Cert Clinical Bioinformatics in their video feedback of the course https://youtu.be/TiIEyEeNiaU (at 2 minutes 15 seconds)

“As an NHS clinician with very little experience of coding, the course and specifically the introduction to programming has a steep learning curve. The modules have all been challenging but the accessibility of tutor support and their proactive approach to supporting students has meant that I’ve never felt lost. As a non-specialist in this field, the course has provided me with **the toolkit to understand the specific role that bioinformatics plays within the NHS**. Whether one goes on undertake further study in this field or not, this PGCert course covers much of the material that a clinician will need familiarity with in the evolving healthcare landscape.”

And

“[…] very grateful for the quality of teaching on the course (across all the modules).”—student on PG Cert Clinical Bioinformatics, who created a reflective presentation on Introduction to Programming (https://www.youtube.com/watch?v=F-YwweY2K-4&feature=youtu.be&hd=1)

Permission was obtained from students to use their statements and videos in publications and for marketing purposes. Videos are publically available on YouTube.

## Discussion

One has to be cautious when introducing such technology into the classroom, especially when running a distance learning/online course if a large part of the course unit is dependent on notebook content. Such notebooks should be tested thoroughly and technical support available for their maintenance and any issues that may arise. Their use may also be more or less problematic with different user groups. Although it is more likely that those from a science, technology, engineering and math (STEM) background will be more comfortable with such tools, we cannot assume that this is necessarily the case. Myths like that of the “digital native” (those born in the age of pervasive digital media) having some special advantages over other generations have been proven to be an unhelpful stereotype [28]. This means that one has to provide adequate support for the use of such tools to ensure their smooth adoption catering for different levels of digital literacy. Support for students with accessibility needs is also a consideration, and where possible, web content should conform to Web Content Accessibility Guidelines (WCAG) [29]. To achieve these aims, it is useful to place the students at the centre of the design process, considering who the target audience is and their needs, the application of the desired learning principles, how they will be presented, and importantly, how this design stands the test of time and is able to be adapted to meet changing needs [30].

For the MIE module (case study 1), we were careful not to assume prior knowledge especially given the diverse nature of the backgrounds and experience of Health Informatics students enrolled on that module. As this was applied to a blended module, we could not make use of standard computer clusters with preloaded software. Most of the students would be accessing the module using their own laptops/devices; therefore, we wanted to avoid the setup issues of downloading and installing a Python distribution (at least initially) until they had gained some familiarity and confidence with coding. We also didn’t want to introduce an IDE at the initial stage of the module or use the console as this is not ideal for writing larger blocks of code. These issues were overcome by remotely hosting the notebooks using cloud services and providing a link for the students to log in via the main university login system. This way, they would have their own secure copy of the notebooks for the module that could be accessed and modified allowing them to rapidly focus on writing and learning to code, rather than all of the peripheral setup requirements and support issues.

In contrast, the approach for the Introduction to Programming module was to provide an authentic and immersive learning journey. The aim was to try to simulate everyday clinical bioinformatics working practices but in a safe learning environment that could give them the space to fail and learn from their mistakes both individually and as a team. This meant students needed to download the notebooks locally and work with different versions using GitHub and work as a team on Slack. The challenge was to provide enough support to help deal with any issues they had with the tools and techniques being taught but enough autonomy so they could develop problem-solving skills much needed in clinical bioinformatics. This balancing act required a lot of resource both at design stage with additional materials for different learner requirements and during the delivery stage.

Supporting modules such as these may require more resources and support than traditional face-to-face modalities. The interactive coding tasks helped the student to gain hands on experience in coding while customising their own class notes, which they can download and keep beyond the duration of the course. Such tasks and interactive elements provided via notebooks uniquely help to move students from a more static learning experience into a more dynamic experience [17]. This can provide a deeper level of immersion in the tasks (for example, exploring a dataset via an interactive plot or applying skills to a practice-based problem of their choosing). In terms of Blooms (revised) taxonomy, this moves students towards the top of higher ordered thinking into creation and production [31].

Careful consideration should also be afforded to the overall aims of the module/unit/course, and technology should be used where appropriate to improve or facilitate learning, rather than be used for novelty purposes. Findings suggest that learning should be the main focus, rather than an aim to be “tech-centric” [32]. If we want to apply digital pedagogy successfully, we need to match each bit of technology with our required pedagogical goals [32]. Essentially, the technology is there to enhance the learning and should be chosen to support the fulfilment of the learning objectives while considering the students’ wider contexts and learning environments. A further consideration is the quality and practices that we impart through such methods. There are calls for journal editors and reviewers to enforce computational reproducibility [33]; however, many scientists use and write code on a regular basis but often lack formal training in good software engineering practices [34]. To embed good practices that students can use in their further research careers, we need to ensure that whenever possible, the content we generate helps to distil these practices. This in turn negates the importance of providing appropriate training for educators themselves.

## Conclusion

The use of Jupyter notebooks on several of our university modules has been positively received by both staff and students who see them as a useful resource for learning to code and communicate research findings and analysis, and in the cases presented, learning the Python programming language specifically. The use of notebooks in such units also gives students an introduction to the notebook environment, which some may go on to use for research purposes later in their career or for the research component of their masters degrees. The use of digital notebooks and other technologies should be carefully evaluated to ensure they add real value to the learning aims and objectives, placing the pedagogic aims of the course at the centre of the process. Given that the use of such tools is becoming more ubiquitous in the bioscience research and scientific education domains, it would be advantageous for academic tutors in such fields to have an awareness and understanding of their application and to consider their use for providing interactive components to computational learning tasks where appropriate.
